# The Effectiveness of Nutritional Interventions on Maternal and Childhood Anaemia in Sindh, Pakistan

**DOI:** 10.3390/nu17233701

**Published:** 2025-11-26

**Authors:** Khizar Ashraf, Michael J. Dibley, Javeria Ikram, Muhammad Umer, Gul Nawaz Khan, Imran A. Chauhadry, Shabina Ariff, Tanvir M. Huda, Sajid B. Soofi

**Affiliations:** 1 Sydney School of Public Health, Faculty of Medicine and Health, The University of Sydney, Sydney, NSW 2006, Australia; khizarashraf@gmail.com; 2 South Western Sydney Local Health District, Liverpool, NSW 2170, Australia; 3 Centre of Excellence in Women & Child Health, The Aga Khan University, Karachi 74800, Pakistan; muhammad.umer@aku.edu (M.U.); shabina.ariff@aku.edu (S.A.)

**Keywords:** nutrition, Pakistan, maternal anaemia, childhood anaemia, behaviour change communication, lipid-based nutrient supplements, programmatic review, WHO anaemia framework

## Abstract

Background/Objectives: In Pakistan, 41.7% of women of reproductive age and 53.7% of children aged 6–59 months are anaemic. This study aimed to evaluate the effectiveness of a nutritional supplementation programme, underpinned with behaviour change communication and implemented through Lady Health Workers (LHWs), in reducing anaemia among pregnant and lactating women as well as 6–59-month-old children. Methods: This study used a quasi-experimental design with an intervention and a control group. A total of 2821 and 2410 pregnant and lactating women and 3397 and 3277 children aged 6–59 months participated in the baseline and endline surveys, respectively. The study areas were matched for demographic and economic characteristics, and both had routine public-sector health and nutrition programmes, including iron–folic acid supplementation during pregnancy. The participants in the intervention group received additional nutritional support. Throughout the pregnancy and in the first six months of exclusive breast feeding, women were provided 5 kg (165 g/day) of wheat soya blend (WSB) per month. Children aged 6–23 months received lipid-based nutrient supplements (LNS), and those aged 24–59 months received micronutrient powder. We applied a difference-in-difference (DID) analysis with kernel propensity score matching to assess the impact on anaemia, a secondary outcome of a stunting prevention programme. Results: Maternal anaemia (both pregnant and breastfeeding women) declined substantially in the intervention areas from 80.4% to 62.6% compared with a smaller reduction in the control areas (80.0% to 72.9%). The DID estimate showed a 10.7-percentage-point (pp) greater decline in maternal anaemia (*p* ≤ 0.001). Both pregnant and lactating women benefited, with DID reductions of 16.7 pp (*p* ≤ 0.001) and 9.4 pp (*p* = 0.005), respectively. The largest gains occurred among women in higher-wealth quintiles, with reductions up to 22.6 pp (*p* ≤ 0.001). In contrast, childhood anaemia showed no overall significant difference between intervention and control areas (DID = −0.7 pp, *p* = 0.73). However, significant improvements were observed among male children (DID = −10.3 pp, *p* = 0.001) and those in higher-wealth quintiles (DID = −15.0 pp, *p* = 0.002). Conclusions: Overall, the findings suggest that LHWs can effectively reduce maternal anaemia when iron–folic acid supplementation is complemented with WSB and targeted behaviour change communication.

## 1. Introduction

Childhood anaemia and maternal anaemia are global public health challenges [1]. The World Health Organization (WHO) defines anaemia as a haemoglobin concentration <11 g/dL in children and pregnant women and <12 g/dL in non-pregnant women. Anaemia may arise from iron or other micronutrient deficiencies, recurrent repeated infections, or underlying genetic disorders [1].

Maternal anaemia increases the risk of preterm birth, postpartum haemorrhage, low birth weight, and pregnancy-related death [2,3]. Low birth weight can lead to neonatal anaemia and developmental delays [4].

Pakistan has high rates of anaemia among pregnant and lactating women and 6–59-month-old children [5,6]. The National Nutrition Survey (NNS) 2011 estimated that 52% of pregnant women in Pakistan were anaemic compared to 61% in Sindh province [7]. According to the NNS 2018, anaemia among women of reproductive age (WRA) declined from 50% in 2011 to 41.7%, with a slightly higher prevalence in rural areas (44.3%). Sindh reported the highest proportion of iron-deficiency anaemia among WRA at 23.8% [8]. A recent systematic review of 16 studies from Pakistan reported a pooled anaemia prevalence of 70.4% during pregnancy [9]. The NNS 2018 also reported that 53.7% of children aged 6–59 months were anaemic, with the prevalence marginally higher in boys (54.2%) than girls (53.1%). Similar to pregnant women, children in rural areas were more anaemic (56.5%) than in urban settings (48.9%) [8].

Anaemia in mothers and children is influenced by multiple socioeconomic factors and high levels of food insecurity, including limited access to essential nutrients [5]. Insufficient nutrient consumption during the initial 1000 days of life leads to anaemia in early childhood [10]. Low fruit and animal protein diets can also lead to micronutrient deficiencies [11,12]. Poor access to and affordability of nutrient-rich foods, such as meat, green leafy vegetables, and fortified products, further reduce iron intake [13]. Cultural factors, such as early marriages and a shorter interval between pregnancies, also increase the risk of maternal anaemia, ultimately affecting foetal development [14,15]. These multifactorial drivers underscore the need for an integrated nutrition and social protection programme. The Benazir Income Support Programme (BISP) is one such example that now includes nutritional supplementation as a core component [16].

Children undergo rapid growth and physiological changes that require adequate nutritional support [17,18]. Infections such as malaria and intestinal parasites further contribute to the increase in prevalence, while poor sanitation and unsafe drinking water lead to recurrent illnesses that impair iron and nutrient absorption [19]. Emerging evidence also links extreme heat waves with substantial increases in childhood anaemia [20]. This could be driven by multiple interacting mechanisms, including physiological stress that worsens nutritional deficiencies, declining food security and dietary quality, and increased risk of infections (such as diarrhoeal illness) that impair iron absorption.

Iron and folic acid (IFA) supplementation is a cost-effective strategy in Pakistan. However, low coverage and adherence are challenges, particularly in food insecure areas [21]. Demographic and Health Survey (DHS) 2013 documents show an increase in daily consumption of IFA supplements from 45% to 65% in 2013 in pregnant women, but gaps persist across Pakistan [22]. Limitations in supply chain, access, training, side effects, and insufficient nutrition education are identified as core reasons for less coverage and adherence [22].

Although Pakistan is an agrarian country, it ranks 84th out of 113 countries on the Global Food Security Index 2022, showcasing widespread vulnerabilities continuing for decades [23,24]. The provision of specialised nutritious food (SNF), developed by the World Food Programme (WFP), during the initial 1000 days can help overcome critical nutrient gaps [11,25,26,27]. Wheat soy blend (WSB) is a mixture of animal-sourced protein, sugar, oil, soybeans, and adjusted micronutrients [28]. Given poor coverage of IFA, it can be considered a critical top up to reduce maternal anaemia. Lipid-based nutrient supplements (LNS) contain micronutrients and essential fatty acids [29,30]. Infants can easily consume LNS, which can bridge nutrient gaps in the absence of any other programmes [29]. Previous studies have shown that these products improve nutritional outcomes [5,26,31].

Pakistan rolled out the National Programme for Primary Health Care and Family Planning in 1994 to bridge the service delivery gap in underserved communities, both in urban and rural areas. LHWs are recruited locally and trained to deliver basic health services, focusing on maternal and child health. They connect the communities to the healthcare system and promote health awareness and access to essential healthcare. Since inception, their roles have evolved and have been used for delivering various preventative public health initiatives successfully [32].

This study evaluated the effectiveness of nutrition interventions delivered through the LHWs in reducing anaemia among pregnant and lactating women and 6–59-month-old children, assessed as a secondary outcome of a stunting prevention programme.

## 2. Methods

### 2.1. Design of the Study

This study is a quasi-experimental design and compares intervention and control groups with similar demographic, ethnic, and economic characteristics using cross-sectional surveys conducted at baseline in year 2014 and endline in year 2018 [17]. The nutrition intervention targeted pregnant and lactating women and 6–59-month-old children [17,33] and was implemented in 29 Union Councils (UCs)/talukas of the Thatta and Sajwal districts, which were classified as food insecure. The control group participants did not receive this intervention. Both groups shared similar baseline characteristics and were selected from within these 29 UCs.

Areas with LHW coverage were selected as intervention sites, whereas those without LHW coverage served as the control. Efforts were made to ensure that all other health services were equally available in both areas. LHWs distributed nutritional supplements and delivered behaviour change communication (BCC), focusing on infant and young child feeding (IYCF) practices, maternal health, nutrition, and hygiene promotion [17]. In this study, we focused on anaemia as a secondary outcome, examining differences in anaemia levels among pregnant and lactating women and children aged 6–59 months between the intervention and control groups.

**Figure 1:**
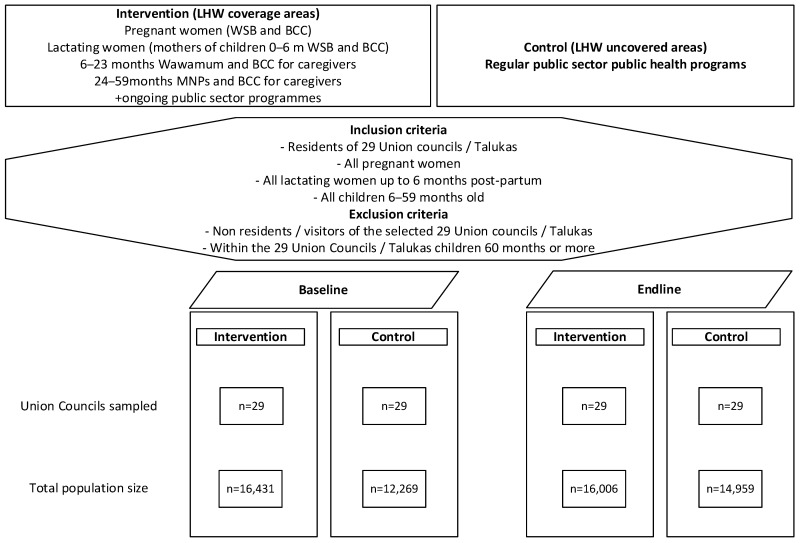
Study participation in the intervention and control groups.

All pregnant and lactating women and 6–59-month-old children were eligible for enrolment (Figure 1). This study followed principles of equity, did not reduce services for the control group, and aimed to generate evidence needed to ultimately benefit the target population at scale.

The eligible women in the intervention group received wheat soy blend (WSB) on a daily basis, children aged 6–23 months received lipid-based nutrient supplements (LNS) a sachet a day, and children aged 24–59 months received multi-micronutrient powder (MNP) on a daily basis along with BCP. The nutritional profiles of the products used are provided in the Supplementary Table S1. The control group did not receive nutrition interventions [17].

### 2.2. Ethics Approval

The Aga Khan University Ethics Review Committee (approval number: 2919-Ped-ERC-14, received on 31 January 2014) and the National Bioethics Committee of Pakistan approved this study. This study was registered at ClinicalTrials.gov (ID: NCT02422953). The study participants provided written and informed consent prior to enrolment.

### 2.3. Study Areas

Due to the high prevalence of malnutrition and micronutrient deficiencies, Thatta and Sajawal districts were selected [17]. Thatta was originally one district at the commencement of this study but was later sub-divided into Thatta and Sajawal.

### 2.4. Collection of Data

Data collection was conducted at baseline in 2014 and endline in 2018 using a standardised field-tested, paper-based questionnaire [17]. Each field team had four female data collectors and a team leader [17]. To ensure uniformity, all team members received a structured six days of training before data collection. The standard operating procedures were followed, and regular field supervision was also undertaken. This is the same methodology that was used in the National Nutrition Surveys, which strengthens the appropriateness and comparability of our approach. The baseline and endline surveys captured information on sociodemographic characteristics, health-seeking behaviour, past intervention exposure, and anthropometric measurements.

Anaemia levels were assessed through spot testing using a HemoCue^®^ Hb 201^+^ System, HemoCue AB, Ängelholm, Sweden [33]. A supervising team conducted data quality assurance [33] and visited 5% of the households to validate the data collection, and the results demonstrated acceptable consistency, indicating satisfactory device and operator performance.

### 2.5. Intervention Procedures

LHWs distributed nutrition supplements. Wheat soy blend (WSB) fortified with micronutrients was provided to PLWs [17]. All PLWs received 5 kg (equivalent to 165 g/day) of WSB every month throughout their pregnancy and the first six months of breastfeeding. Children aged 6–23 months were given lipid-based nutrient supplements (LNS) branded as Wawamum (Table 1), whereas children aged 24–59 months received multiple micronutrient powders (MNP) (Table 1). PLW and children aged 6–59 months in the control group received standard routine care.

### 2.6. Sample Size

The calculation of sample size has been published previously [17]. It was based on this study’s primary objective of achieving a 10% reduction in stunting prevalence over four years of implementation [17]. It was increased by 15% considering dropouts, data errors, and refusals. The sample size provided sufficient power (90%) to assess a 10% difference between the intervention and the control groups. Statistical significance was set at *p* ≤ 0·05. LHW family household records were used to identify eligible participants randomly. All households with pregnant and lactating women or 6–59-month-old children were eligible for inclusion in both surveys.

### 2.7. Statistical Analysis

STATA version 18 was used for data analysis. We analysed household characteristics for all participants and constructed wealth quintiles using principal component factors (PCF) for extraction of maximum variance and also data reduction [33,34]. This includes 33 variables: ownership of land, livestock, assets, household construction, and sanitation [17].

We used difference-in-differences (DID) analyses for repeated cross-sectional surveys (baseline and endline) to examine the program’s impact on child outcomes by utilising the value of the propensity score [35,36,37]. Details are already published [17].

## 3. Results

We analysed 2821 pregnant and lactating women (1536 intervention and 1285 control groups) at baseline and 2410 (1650 intervention and 760 control group) at the endline. We also analysed 3397 children aged 6–5.9 months at the baseline survey compared to 3277 children at the endline survey. The subgroup of children 6–23 months old included 1506 children (849 intervention group and 657 control group) at the baseline survey and 1451 children (740 intervention group and 711 control group) at the endline survey. For subgroup 24–59 months old, there were 1891 children (983 intervention group and 908 control) at the baseline survey and 1826 children (910 intervention group and 916 control) at the endline survey (Table 1).

### 3.1. Characteristics at the Baseline Survey

The average household density in the intervention cohort was 0.9 more than in the control (Table 1), which was statistically significant (*p* ≤ 0.001). At both baseline and endline, the control group had lower levels of maternal education than the intervention group. The baseline differences were adjusted in subsequent analyses.

### 3.2. Characteristics at Endline Survey

The average household density was similar between the intervention and control groups (7.3 persons per household, *p* = 0.689). There were slightly more female children in the intervention group, which was not the same in the control group. More mothers in the control group had no education compared to the intervention group.

### 3.3. Intervention Effects on the Prevalence of Maternal Anaemia

Table 2 shows the differential changes in maternal anaemia between the intervention and control groups over the four years of the implementation period. Overall, the intervention produced a significant reduction in maternal anaemia, with a difference-in-differences (DID) estimate of −10.7 percentage points, (*p* ≤ 0.001). Among pregnant women, the reduction was more pronounced (DID = −16.7 pp, *p* ≤ 0.001), while lactating women also showed a significant improvement (DID = −9.4 pp; *p* = 0.005). The effect was significant among mothers with no education (*p* = 0.005) and those with primary-level education (*p* = 0.003). We observed substantial variation in the effect of the intervention on maternal anaemia among the different wealth groups. The difference was most pronounced in the two wealthiest quintiles.

### 3.4. Intervention Effects on the Prevalence of Childhood Anaemia

Table 3 shows that the intervention had no impact on childhood anaemia (6–59 months) (DID = −0.7 pp, *p* = 0.733). Subgroup analysis showed no significant impact in anaemia among children aged 6–23 months (DID = −1.4 pp, *p* = 0.636) or those aged 24–59 months (DID = −2.9 pp, *p* = 0.298). However, the intervention significantly reduced anaemia among male children (DID = −10.3 pp, *p* = 0.001), while no effect was observed among females (DID = 1.7 pp, *p* = 0.570). The largest decline was seen among children in the highest wealth quintile, where anaemia prevalence fell by −15 pp (*p* = 0.002).

## 4. Discussion

The DID analysis showed that the intervention significantly reduced maternal anaemia compared to the control group. This impact was evident in both pregnant and lactating women, with the largest gains among women in higher wealth quintiles. Although the intervention did not significantly impact overall childhood anaemia, significant improvements were observed among male children and those from higher wealth quintiles. As previously reported, the intervention substantially reduced childhood stunting [17]. This suggests that, while maternal intervention has clear benefits, its translation to reducing childhood anaemia is limited.

Strengthening maternal and child healthcare services is crucial for addressing anaemia in Pakistan. Increasing antenatal care (ANC) coverage and improving the quality of child and maternal health services can support the early detection and treatment of anaemia [38]. IFA supplementation reduces anaemia in pregnant and lactating women [39]. Various studies suggest that adherence to a daily intake of IFA remains a challenge [40]. The alarmingly high anaemia levels in Thatta, Sajawal, and across Sindh province suggest that routine iron–folic acid supplementation programs are not optimally effective, which could be due to the low coverage and adherence, particularly low in rural areas of Sindh [22]. The WHO recommends daily supplementation for all pregnant women with 30–60 mg of iron and 0.4 mg of folic acid [41,42]. WSB provides an additional 13.9 mg iron and 0.1 mg of folic acid top up, which could bridge the nutrient gap for some mothers, particularly from higher wealth quintiles and those experiencing challenges with daily IFA supplementation. This could be due to comparatively low levels of food insecurity and better dietary intake. WSB had a good acceptance in the participating women, which was also evident in the RCT, demonstrating the impact of intervention [5].

The risk of anaemia begins in utero [43], as children born to anaemic mothers can be iron deficient and anaemic early in life. Iron stores become depleted during rapid red blood cell production in infancy [44], especially in high risk and infectious environments [45], which is possible in rural areas like Thatta and Sajawal districts due to poor sanitation. The 24–59-month-old children undergo rapid growth, requiring more iron for red blood cells [46]. If sanitation is poor, they are likely to acquire infections that can cause iron deficiency [47]. Deworming is a recommended strategy for children along with supplementation [45]. Studies suggest that low doses of iron supplementation without anthelmintic treatment do not improve anaemia [45].

The iron supplementation in the LNS for children aged 6–24 months provided 5 mg daily, which is below the WHO-recommended 10–12.5 mg of elemental iron for settings where childhood anaemia prevalence exceeds 40% [43]. Similarly, the MNP for children aged 24–59 months contained 10 mg iron, also lower than the WHO-recommended 30 mg of elemental iron for high-anaemia settings [43]. Given that no other supplementation programme was taking place in Thatta and Sajawal, the LNS could not optimally bridge the nutrient gap.

The RCT performed in Thatta and Sajawal districts documented positive outcomes [5,33]. The operational challenges in implementation through LHWs can have a reduced impact [17]. We also observed an interrupted supply chain of nutritious products, less prioritisation by lady health workers, and sharing among family members. The quality of BCC is also important. It is only effective if we can identify barriers and then design education sessions focusing on enablers. Nutrition education without focusing on enablers may not be beneficial [48]. Lady health workers play a vital role in BCC towards mothers and families about the importance of iron-rich diets, supplementation, and timely healthcare-seeking behaviour. Strengthening the capacity and coverage of LHWs and primary care services, particularly in rural and underserved areas, can ensure that more women and children receive essential nutrition and health services.

The lack of impact on childhood anaemia may be due to several factors: LNS may not have been sufficient to address underlying iron deficiency; gaps in routine public-sector deworming campaigns could have reduced the effectiveness of nutrient absorption; and sharing of supplements among siblings may have diluted the intended benefits. This programme relied heavily on the public sector’s capacity to add nutrition interventions during routine service delivery. Another limitation is the unavailability of infectious disease data in the implementation areas, which could explain underlying infection rates. Adherence to the daily consumption of LNS and micronutrient sachets was also challenging in the beginning, which was later managed by redesigned and attractive packaging. The initial English language labelling had resemblance to contraceptive products, which was resisted by the community. We also believe that households in the least wealthy quintiles were possibly hard to reach and suffered the most supply chain delays. Another limitation is to identify children and pregnant and lactating women who are severely anaemic and treat them before focusing exclusively on preventative supplementation. Accommodating this will definitely benefit the outcomes.

In addition, the highest wealth quintiles among pregnant and lactating women and children showed an impact, illustrating that nutrient gaps were wider in the lower wealth quintiles, which requires nutrition equity to be considered along with the root causes [49]. This suggests that, in designing population-wide interventions, elements of equity in terms of nutrient gaps should be considered. A co-design approach may help in future programming. Another finding was that the improvement was significant for boys but not for girls, possibly due to gender preference observed in the local context [49,50]. We also need to note that, despite matching control and intervention areas on key contextual factors, residual unmeasured differences (including those related to IFA supply chain, training, and health-seeking behaviours) may still have influenced the outcomes. In this study, the selection of these areas was based on predefined program implementation boundaries rather than researcher choice, meaning it does not introduce selection bias. The comparison reflects the real-world distribution of LHW services rather than selective sampling.

Previous studies have provided evidence that supplementation and behaviour change communication (BCC) encourage dietary diversity [36]. These interventions attempted to bridge the nutrient gap through supplementation reinforced by BCC through an existing LHW program. The product was sourced through local supply chains; therefore, sustainability may not be an issue. This intervention may be considered in the national and provincial nutrition and maternal, neonatal, and childhood strategies, where it becomes part of the LHW’s routine antenatal care and outreach activities. The supply chain can be sustained by public sector procurement for disadvantaged communities (as various programs such as Ehsaas Nashonuma and Benazir’s income support program exist) [51]. We also need to consider that the nutrient gap is wider at the population level and that this intervention was most effective at the highest wealth quintiles, a pattern similar to improvements in stunting [17]. There may be significant nutrient gaps in local diets for various reasons (including extreme poverty and rising food costs), which also impact complementary feeding at home and dietary adequacy, and this intervention may not be fully sufficient [17,36]. Similarly, the preference for boys over girls might be another reason why DID was significant in male children. The significant impact on maternal and childhood anaemia in the highest wealth quintiles and boys supports the exploration of further scale-up.

This intervention faced challenges comparable to other trials, in which low-dose supplementation alone may not be effective for childhood anaemia [45]. There is evidence that a reduction in micronutrient and nutritional deficiencies is multisectoral, and underlying factors, such as the social and political situation, economic factors, and sanitation conditions, cannot be ignored [52]. The WHO framework for anaemia control also recommends multiple domain-tailored solutions addressing underlying risk factors and fundamental drivers [53].

## 5. Conclusions

Our findings suggest that LHWs can play a vital role in combating maternal anaemia by providing additional WSB supplementation on top of the recommended IFA dose and through effective behaviour change communication. This will likely bridge the nutrient gap due to limitations of IFA uptake and coverage. In contrast, childhood anaemia showed limited overall improvement, with meaningful gains observed mainly among male children and those from better-off households.

These findings suggest that this intervention may not optimally reduce anaemia in 6–59-month-old children and that additional targeted strategies are needed, especially for socioeconomically disadvantaged groups. Thus, for healthcare policy makers, it is important to prioritise and finance policies that support universal access to nutrition services by establishing local supply chains and a trained workforce and embedding nutrition screening and counselling within maternal and child health services. For clinical practice, integrating nutrition in maternal child health services can improve health outcomes.

## Figures and Tables

**Table 1:** Characteristics of study participants.

Characteristics	Baseline				Endline			
	Intervention		Control		Intervention		Control	
	n	%	n	%	n	%	n	%
Density of household	7.1		6.2		7.3		7.3	
**Women** (pregnant and lactating)	1536		1285		1650		760	
**Children aged 6–59 months**	1832		1565		1650		1627	
**Gender**								
Male	950	51.9	800	51.1	807	48.9	867	53.3
Female	882	48.1	765	48.9	843	51.1	760	46.7
**Age**	1832		1565		1650		1627	
6–23 months	849	46.3	657	42.0	740	44.8	711	43.7
24–59 months	983	53.7	908	58.0	910	55.2	916	56.3
**Maternal education**								
None	1469	80.2	1536	98.1	1368	82.9	1517	93.2
Primary or middle	267	14.6	27	1.7	206	12.5	90	5.5
Secondary or higher	96	5.2	2	0.1	76	4.6	20	1.2
**Household wealth quintiles**								
Lowest	187	10.2	509	32.5	223	13.5	449	27.6
Second	266	14.5	405	25.9	262	15.9	397	24.4
Middle	353	19.3	326	20.8	321	19.5	343	21.1
Fourth	446	24.3	251	16.0	402	24.4	251	15.4
Highest	580	31.7	74	4.7	442	26.8	187	11.5

**Table 2:** Effects on the prevalence of maternal anaemia.

	Maternal Anaemia %									
	Baseline				Endline					
	Intervention	Control	Difference	*p*-Value	Intervention	Control	Difference	*p*-Value	DID	*p*-Value
**Maternal Anaemia**	80.4	80.0	0.4	0.808	62.6	72.9	−10.3	<0.001	−10.7	<0.001
Maternal Anaemia (lactating)	78.0	78.7	−0.7	0.750	59.2	69.3	−10.1	<0.001	−9.4	0.005
Maternal Anaemia (pregnant)	84.3	79.3	5.0	0.048	67.1	78.8	−11.7	<0.001	−16.7	<0.001
**Maternal Education**										
None	81.6	84	−2.4	0.167	64.3	73.9	−9.6	<0.001	−7.2	0.005
Primary or Middle	76.6	65.7	10.9	0.040	58.7	72.8	−14.1	0.027	−25	0.003
**by Wealth Quintiles**										
Lowest	87.2	87.6	−0.4	0.901	65.5	75.2	−9.7	0.022	−9.3	0.105
Second	83.5	84.5	−1.0	0.771	66.0	81.3	−15.3	<0.001	−14.3	0.012
Middle	80.6	86.7	−6.1	0.074	66.7	68.3	−1.6	0.701	4.5	0.420
Fourth	79.0	77.7	1.3	0.709	64.4	82.6	−18.2	<0.001	−19.5	<0.001
Highest	77.8	67.5	10.3	0.004	54.5	66.8	−12.3	0.003	−22.6	<0.001

**Table 3:** Effects on the prevalence of childhood anaemia.

	Childhood Anaemia %									
	Baseline				Endline					
	Intervention	Control	Difference	*p*-Value	Intervention	Control	Difference	*p*-Value	DID	*p*-Value
**Anaemia**										
6–59 months	88.6	88.2	0.4	0.766	79.5	79.8	−0.3	0.846	−0.7	0.733
6–23 months	90.1	84.4	5.7	0.004	84.3	80.0	4.3	0.059	−1.4	0.636
24–59 months	87.3	88.8	−1.5	0.431	75.7	80.1	−4.4	0.042	−2.9	0.298
Males	88.9	79.3	9.6	<0.001	79.2	79.9	−0.7	0.742	−10.3	0.001
Females	88.2	90.1	−1.9	0.323	79.9	80.1	−0.2	0.914	1.7	0.570
**by Maternal Education**										
None	88.9	87.8	1.1	0.449	79.7	81.4	−1.7	0.329	−2.8	0.217
Primary or Middle	88.6	76.7	11.9	0.009	80.8	71.7	9.1	0.098	−2.8	0.687
**by Wealth Quintiles**										
Lowest	83.4	90.3	−6.9	0.045	79.7	85.1	−5.4	0.160	1.5	0.770
Second	90.6	89.8	0.8	0.790	72.0	85.3	−13.3	0.001	−14.1	0.005
Middle	92.9	89	3.9	0.145	84.0	84.5	−0.5	0.881	−4.4	0.308
Fourth	88.0	88.2	−0.2	0.949	80.5	77.4	3.1	0.361	3.3	0.468
Highest	86.6	68	18.6	<0.001	79.7	76.1	3.6	0.324	−15.0	0.002

## Data Availability

Restrictions apply to the availability of these data. Data are available on request with the permission of the corresponding author of Aga Khan University, Pakistan.

## Supplementary Materials

The following supporting information can be downloaded at: https://www.mdpi.com/article/10.3390/nu17233701/s1, Table S1: Contents of supplements.

## References

[1] WHO (2021). WHO Global Anaemia Estimates.

[2] Edelson P.K., Cao D., James K.E., Ngonzi J., Roberts D.J., Bebell L.M., Boatin A.A. (2023). Maternal anaemia is associated with adverse maternal and neonatal outcomes in Mbarara, Uganda. J. Matern. Fetal Neonatal Med..

[3] Wang R., Xu S., Hao X., Jin X., Pan D., Xia H., Liao W., Yang L., Wang S. (2025). Anaemia during pregnancy and adverse pregnancy outcomes: A systematic review and meta-analysis of cohort studies. Front. Glob. Womens Health.

[4] Hilaire M., Andrianou X.D., Lenglet A., Ariti C., Charles K., Buitenhuis S., Van Brusselen D., Roggeveen H., Ledger E., Denat R.S. (2021). Growth and neurodevelopment in low birth weight versus normal birth weight infants from birth to 24 months, born in an obstetric emergency hospital in Haiti, a prospective cohort study. BMC Pediatr..

[5] Soofi S.B., Khan G.N., Ariff S., Ihtesham Y., Tanimoune M., Rizvi A., Sajid M., Garzon C., de Pee S., Bhutta Z.A. (2022). Effectiveness of nutritional supplementation during the first 1000-days of life to reduce child undernutrition: A cluster randomized controlled trial in Pakistan. Lancet Reg. Health Southeast Asia.

[6] Harding K.L., Aguayo V.M., Namirembe G., Webb P. (2018). Determinants of anaemia among women and children in Nepal and Pakistan: An analysis of recent national survey data. Matern. Child. Nutr..

[7] Government of Pakistan (2011). National Nutrition Survey.

[8] UNICEF (2019). National Nutrition Survey 2018, Key Findings Report.

[9] Mahar B., Shah T., Shaikh K., Shaikh S.N., Uqaili A.A., Memon K.N., Warsi J., Mangi R., Aliyu S., Abbas Q. (2024). Uncovering the hidden health burden: A systematic review and meta-analysis of iron deficiency anaemia among adolescents, and pregnant women in Pakistan. J. Health Popul. Nutr..

[10] Cusick S.E., Georgieff M.K. (2016). The Role of Nutrition in Brain Development: The Golden Opportunity of the “First 1000 Days”. J Pediatr.

[11] Dewey K.G. (2016). Reducing stunting by improving maternal, infant and young child nutrition in regions such as South Asia: Evidence, challenges and opportunities. Matern. Child. Nutr..

[12] Dewey K.G., Adu-Afarwuah S. (2008). Systematic review of the efficacy and effectiveness of complementary feeding interventions in developing countries. Matern. Child. Nutr..

[13] Osei Bonsu E., Addo I.Y., Boadi C., Boadu E.F., Okeke S.R. (2024). Determinants of iron-rich food deficiency among children under 5 years in sub-Saharan Africa: A comprehensive analysis of Demographic and Health Surveys. BMJ Open.

[14] Tiruneh F.N., Tenagashaw M.W., Asres D.T., Cherie H.A. (2021). Associations of early marriage and early childbearing with anaemia among adolescent girls in Ethiopia: A multilevel analysis of nationwide survey. Arch. Public Health.

[15] Addis Alene K., Mohamed Dohe A. (2014). Prevalence of Anemia and Associated Factors among Pregnant Women in an Urban Area of Eastern Ethiopia. Anaemia.

[16] BISP Benazir Nashonuma. https://bisp.gov.pk/Detail/YjAyMjI5ZDQtMTVkOC00YTNlLWE5NjctMjA1NTYwN2JhOTE3.

[17] Ashraf K., Huda T.M., Ikram J., Ariff S., Sajid M., Khan G.N., Umer M., Ahmed I., Dibley M.J., Soofi S.B. (2024). The Effectiveness of Nutritional Interventions Implemented through Lady Health Workers on the Reduction of Stunting in Children under 5 in Pakistan: The Difference-in-Difference Analysis. Nutrients.

[18] Beluska-Turkan K., Korczak R., Hartell B., Moskal K., Maukonen J., Alexander D.E., Salem N., Harkness L., Ayad W., Szaro J. (2019). Nutritional Gaps and Supplementation in the First 1000 Days. Nutrients.

[19] van Cooten M.H., Bilal S.M., Gebremedhin S., Spigt M. (2019). The association between acute malnutrition and water, sanitation, and hygiene among children aged 6-59 months in rural Ethiopia. Matern. Child. Nutr..

[20] Fanzo J., Carducci B. (2025). Anaemia in a time of climate crisis. Lancet Haematol..

[21] UNICEF (2022). Iron Folic Acid (IFA) Bottleneck Analysis Report Pakistan.

[22] National Institute of Population Studies (NIPS) (2019). Pakistan Demographic and Health Survey 2017–18.

[23] Khan M., Shah A. (2011). Food Insecurity in Pakistan: Causes and Policy Response. J. Agric. Environ. Ethics.

[24] (2022). G.F.S.I. https://impact.economist.com/sustainability/project/food-security-index/.

[25] Adu-Afarwuah S., Lartey A., Dewey K.G. (2017). Meeting nutritional needs in the first 1000 days: A place for small-quantity lipid-based nutrient supplements. Ann. N. Y. Acad. Sci..

[26] Adu-Afarwuah S., Lartey A., Okronipa H., Ashorn P., Peerson J.M., Arimond M., Ashorn U., Zeilani M., Vosti S., Dewey K.G. (2016). Small-quantity, lipid-based nutrient supplements provided to women during pregnancy and 6 mo postpartum and to their infants from 6 mo of age increase the mean attained length of 18-mo-old children in semi-urban Ghana: A randomized controlled trial. Am. J. Clin. Nutr..

[27] Ceesay S.M., Prentice A.M., Cole T.J., Foord F., Weaver L.T., Poskitt E.M., Whitehead R.G. (1997). Effects on birth weight and perinatal mortality of maternal dietary supplements in rural Gambia: 5 year randomised controlled trial. Bmj.

[28] Mridha M.K., Matias S.L., Chaparro C.M., Paul R.R., Hussain S., Vosti S.A., Harding K.L., Cummins J.R., Day L.T., Saha S.L. (2016). Lipid-based nutrient supplements for pregnant women reduce newborn stunting in a cluster-randomized controlled effectiveness trial in Bangladesh. Am. J. Clin. Nutr..

[29] Lazzerini M., Rubert L., Pani P. (2013). Specially formulated foods for treating children with moderate acute malnutrition in low- and middle-income countries. Cochrane Database Syst. Rev..

[30] Lesorogol C., Jean-Louis S., Green J., Iannotti L. (2015). Preventative lipid-based nutrient supplements (LNS) and young child feeding practices: Findings from qualitative research in Haiti. Matern. Child. Nutr..

[31] Siega-Riz A.M., Estrada Del Campo Y., Kinlaw A., Reinhart G.A., Allen L.H., Shahab-Ferdows S., Heck J., Suchindran C.M., Bentley M.E. (2014). Effect of supplementation with a lipid-based nutrient supplement on the micronutrient status of children aged 6–18 months living in the rural region of Intibucá, Honduras. Paediatr. Perinat. Epidemiol..

[32] Wazir M.S., Shaikh B.T., Ahmed A. (2013). National program for family planning and primary health care Pakistan: A SWOT analysis. Reprod. Health.

[33] Kureishy S., Khan G.N., Arrif S., Ashraf K., Cespedes A., Habib M.A., Hussain I., Ullah A., Turab A., Ahmed I. (2017). A mixed methods study to assess the effectiveness of food-based interventions to prevent stunting among children under-five years in Districts Thatta and Sujawal, Sindh Province, Pakistan: Study protocol. BMC Public Health.

[34] Ahmed S., Mehedi Hasan M., Ahmed W., Atiqul Hoque Chowdhury M. (2013). Socio-economic Inequity of Malnutrition among Under-Five Children and Women at Reproductive Age in Bangladesh. World J. Nutr. Health.

[35] Wing C., Simon K., Bello-Gomez R.A. (2018). Designing Difference in Difference Studies: Best Practices for Public Health Policy Research. Annu. Rev. Public Health.

[36] Christian P., Hurley K.M., Phuka J., Kang Y., Ruel-Bergeron J., Buckland A.J., Mitra M., Wu L., Klemm R., West K.P. (2020). Impact Evaluation of a Comprehensive Nutrition Program for Reducing Stunting in Children Aged 6-23 Months in Rural Malawi. J. Nutr..

[37] Abadie A. (2018). Difference-in-Difference Estimators. The New Palgrave Dictionary of Economics.

[38] Saapiire F., Dogoli R., Mahama S. (2022). Adequacy of antenatal care services utilisation and its effect on anaemia in pregnancy. J. Nutr. Sci..

[39] Allen L.H. (2005). Multiple micronutrients in pregnancy and lactation: An overview2. Am. J. Clin. Nutr..

[40] Banerjee A., Athalye S., Shingade P., Khargekar V., Mahajan N., Madkaikar M., Khargekar N. (2024). Efficacy of daily versus intermittent oral iron supplementation for prevention of anaemia among pregnant women: A systematic review and meta-analysis. eClinicalMedicine.

[41] WHO Daily Iron and Folic Acid Supplementation During Pregnancy. https://www.who.int/tools/elena/interventions/daily-iron-pregnancy.

[42] Peña-Rosas J.P., De-Regil L.M., Garcia-Casal M.N., Dowswell T. (2015). Daily oral iron supplementation during pregnancy. Cochrane Database Syst. Rev..

[43] WHO (2016). Daily Iron Supplementation in Infants and Children.

[44] Chaparro C.M. (2008). Setting the stage for child health and development: Prevention of iron deficiency in early infancy. J. Nutr..

[45] Stoltzfus R.J., Chway H.M., Montresor A., Tielsch J.M., Jape J.K., Albonico M., Savioli L. (2004). Low dose daily iron supplementation improves iron status and appetite but not anemia, whereas quarterly anthelminthic treatment improves growth, appetite and anemia in Zanzibari preschool children. J. Nutr..

[46] Wharton B.A. (1999). Iron deficiency in children: Detection and prevention. Br. J. Haematol..

[47] Hotez P.J., Brooker S., Bethony J.M., Bottazzi M.E., Loukas A., Xiao S. (2004). Hookworm infection. N. Engl. J. Med..

[48] Fabrizio C.S., van Liere M., Pelto G. (2014). Identifying determinants of effective complementary feeding behaviour change interventions in developing countries. Matern. Child Nutr..

[49] Basu A.M. (1993). How pervasive are sex differentials in childhood nutritional levels in south Asia?. Soc. Biol..

[50] Nuruddin R., Hadden W.C. (2015). Are pre-school girls more likely to be under-nourished in rural Thatta, Pakistan?-a cross-sectional study. Int. J. Equity Health.

[51] Sriram S.A.-O., Naz L. (2025). Inequality of opportunity in child nutrition in Pakistan. PLoS ONE.

[52] Roba A.A., Assefa N., Dessie Y., Tolera A., Teji K., Elena H., Bliznashka L., Fawzi W. (2021). Prevalence and determinants of concurrent wasting and stunting and other indicators of malnutrition among children 6–59 months old in Kersa, Ethiopia. Matern. Child. Nutr..

[53] Atkinson S.H., Suchdev P.S., Bode M., Carducci B., Cerami C., Mwangi M.N., Namaste S., Winichagoon P., Leung S., Mutua A.M. (2025). Getting back on track to meet global anaemia reduction targets: A Lancet Haematology Commission. Lancet Haematol..

