# Supplementary material for: The Effectiveness of Nutritional Interventions on Maternal and Childhood Anaemia in Sindh, Pakistan
Source: Nutrients. 2025 Nov 26;17(23):3701. doi: 10.3390/nu17233701 (PMC12694230; doi:10.3390/nu17233701)
Supplement: Supplementary file 1 [file nutrients-17-03701-s001.zip › nutrients-3974617-supplementary.pdf]

Supplementary Materials:

Supplementary table S1

Contents of supplements	Wheat Soya Blend (WSB)	WAWAMUM (Per 50 G)	MNP (Per 1 G)
Daily ration (g/person/day)	167	50	On alternate day
Energy (kcal)	633	255	-
Protein (g)	29.1	5.5	-
Fat (g)	10.2	13	-
Calcium (mg)	683	267.5	-
Iron (mg)	13.9	5	10
Iodine (μg)	67	50	90
Vitamin A (μg RE)	842	275	400
Thiamine B1 (mg)	0.66	0.5	-
Riboflavin Vitamin B2(mg)	1.03	1.05	-
Niacin (mg NE)	15.3	6.5	6
Vitamin C (mg)	168.9	30	30
Pantothenic VitB5 (mg)	3.4	2	-
Vitamin B6 (mg)	1.8	0.9	0.5
Vitamin B7 (μg)	-	30	-
Folic Acid (μg)	100	-	902
Vitamin B12 (μg)	3	1.35	0.9
Vitamin D (μg)	10	7.5	-
Vitamin E(mg)	15.8	8	-
Vitamin K (μg)	-	13.5	-
Cu (mg)	-	0.7	-
Magnesium (mg)	-	75	-
Manganese (mg)	-	0.6	-
Phosphorus (mg)	-	225	-
Potassium (mg)	-	450	-
Selenium (μg)	49.3	10	17
Na (mg)	-	135	-
Zn (mg)	-	5.5	-
Vitamin D3 (μg)	-	-	5
Vitamin E (mg)	-	-	5

**Vitamin B1 (mg)**	**-**	**-**	**0.5**
**Vitamin B2 (mg)**	**-**	**-**	**0.5**
**Zinc (mg)**	**11.2**	**-**	**4.1**
**Copper (mg)**	**0.6**	**-**	**0.56**
**Folate (μg)**	**288**	**165**	**-**
**Dry skimmed milk protein**	**-**	**1.8**	**-**
**ω-3 fatty acids**	**-**	**0.15**	**-**
**ω-6 fatty acid**	**-**	**1.3**	**-**
